# Fabrication of a Low Cost Superhydrophobic Substrate for Surface Enhanced Laser-Induced Breakdown Spectroscopy and Its Utility through Identification of Electrolyte Variation for Oral Cancer Detection

**DOI:** 10.1021/acsbiomaterials.3c01275

**Published:** 2024-01-16

**Authors:** Keerthi K, Sajan Daniel George, Ravikiran Ongole, Unnikrishnan V K

**Affiliations:** † Department of Atomic and Molecular Physics, Manipal Academy of Higher Education, Manipal-576104, India; ‡ Centre for Applied Nanosciences, Department of Atomic and Molecular Physics, Manipal Academy of Higher Education, Manipal-576104, India; § Department of Oral Medicine and Radiology, Manipal College of Dental Sciences, Manipal Academy of Higher Education, Mangalore− 575001, India; ∥ Centre of Excellence for Biophotonics, Department of Atomic and Molecular Physics, Manipal Academy of Higher Education, Manipal-576104, India

**Keywords:** SELIBS, superhydrophobic PDMS, nanostructured material, oral cancer, biomedical sensing

## Abstract

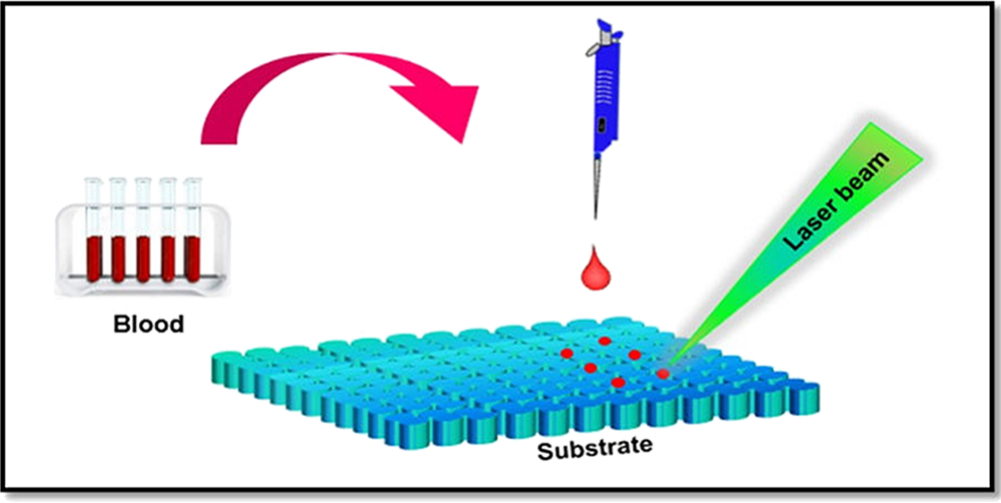

Ultratrace elemental detections from a limited volume of samples can offer significant benefits in biomedical fields. However, it can be challenging to concentrate the particles being analyzed in a small area to improve the accuracy of detection. Ring-like deposits on the edges of colloidal droplets are a vexing problem in many applications. Herein, we report ultratrace elemental detection using a superhydrophobic surface-enhanced laser-induced breakdown spectroscopy (SELIBS) substrate fabricated by laser ablation followed by a soft lithography technique. In this work, the SELIBS spectra on a superhydrophobic polydimethylsiloxane (PDMS) substrate replicated from a laser-patterned master Teflon substrate are investigated. This work highlights the application of this newly created superhydrophobic substrate for detecting trace elements in body fluids using SELIBS. The developed PDMS substrate was successfully adopted to investigate the electrolyte variation in serum samples of oral cancer patients and normal volunteers. Principal component analysis (PCA) and match-no-match analysis were used to distinguish the elemental variation in cancer and control groups.

## Introduction

Laser-induced breakdown spectroscopy (LIBS) is an effective tool for material characterization by virtue of its ability to provide multielemental analysis.^1,2^ Detection of ultratrace elements from a limited volume of samples can provide significant and unprecedented benefits in the biomedical and environmental fields.^3−5^ It is worth noting that extensive research has been undertaken on the analysis of biological samples such as teeth, bones, kidney stones, etc.^6−8^ using a laser-induced breakdown spectroscopy (LIBS) technique. However, fewer studies have focused on body fluid analysis such as serum, saliva, tear etc. due to the inherent challenges associated with liquid LIBS analysis.^9,10^ However, recent studies have shown the ability of the LIBS technique to detect ultratrace elements in biological fluids. Though many researchers have proposed interesting sampling approaches for liquid LIBS analysis,^11−14^ it has not been recognized as an efficient body fluid element detector to date. This is due to multiple drawbacks associated with the technique, including sample preparation, expensive substrates, and limited sample volumes.^15^

Unfortunately, the benefit of LIBS in liquid samples is limited by its uniformity and poor reproducibility due to the dispersion of analyte particles in a large area of the substrate.^16−18^ Despite the success of LIBS in detecting analyte particles in ultradiluted solutions, the concentration of analyte particles in a small area is still highly challenging. While various efforts have been made to overcome these problems, it remains a formidable task to develop substrates for surface-enhanced laser-induced breakdown spectroscopy studies (SELIBS) that facilitate high-density uniform deposition of the analyte in a small probing region.

Recently, the superhydrophobic substrate was emerged as a better alternative to be an efficient tool for addressing these problems.^19−21^ Using the SELIBS technique, a droplet of ultradiluted solution evaporated on the superhydrophobic surface can enrich analyte particles and detected in a sensitive area. The concentration enrichment by the superhydrophobic surface greatly enhances the detection limit of SELIBS over conventional LIBS technique, which are usually hydrophilic. In previous works, Dong et al.^19^ demonstrated a simple method for the preparation of hydrophobic substrate using commercial products. Wu et al. fabricated biomimic surface microdevices with complex structured microchannels by hydrogel micropatterning coupled with liquid molding.^20^ Niu et al. reported a laser pretreated metallic substrate for trace elemental detection in standard solution and river water samples.^22^ Previously, laser patterning was used to create SELIBS substrates that had an array of silicon cones with nanoscale decorations like nanodots. Despite the fact that this technique achieved a better detection limit, it relied on laser ablation for each substrate production, which is a time-consuming and expensive process. Although several methods were attempted to fabricate the SELIBS substrate, most of these techniques demand high operating costs and are not scalable.

Generally, SELIBS substrates are still a long way from being widely used in industry due to their complicated fabrication and high cost. Hence, efforts are being made to develop alternative approaches for fabricating the SELIBS substrate inexpensively. Herein, a superhydrophobic surface was prepared by laser patterning followed by a soft lithography technique. Compared to the previously developed nanoparticle-enhanced LIBS technique, the superhydrophobic nature of the substrate can concentrate analyte particles into a small area, thus significantly enhancing the LIBS intensity. In the present study, the superhydrophobic substrate is fabricated via a two-step process by replicating the laser-patterned master structure, which is free of complex experimental procedures and easy to use. We need to make only one master pattern structure, and using that we can replicate multiple number of substrates, which in turn is cost-effective. This method allows the production of highly hierarchical microscale structures in a single step, eliminating the need for complex photolithography procedures. Due to its extremely low adhesion properties, the contact line of the solution droplet can move freely without experiencing a pinning effect. Consequently, the area after evaporation can be minimized to a small hotspot on PDMS as compared to bare substrates. Notably, this reduction in evaporation area results in an increase in the concentrating effect as well as LIBS signal enhancement.

Although the role of molecular mechanisms in malignancies is well-established, cancer continues to be the second largest reason for fatality in developing nations. Oral cancer stands at the eighth position in malignancies across the world. Cancer patients routinely suffer from electrolyte imbalance relating to variations in serum sodium, potassium, calcium, and magnesium.^27,28^ Generally, such variations are asymptomatic and hence are easily ignored during clinical examination. Nevertheless, they can occasionally be related to clinical signs, which may exacerbate medical emergency. Literature shows correlation between electrolytic disorders and cancer, such as deteriorating quality of life and functioning condition, low response to cancer therapy and treatment delays, insignificant results, and low survival rate.^29^ Cancer pathophysiology, cancer therapy, existing systemic disorders, or other treatments can have an impact on electrolyte disorders. They are of multifactorial origin, may be secondary, and accountable for multiorgan dysfunction. A timely improvement of electrolyte disorders is frequently related to an improved prognosis. Hence, a rising awareness about electrolyte disturbances can be found in databases and clinical investigations. Whole blood and serum are commonly used as samples for the analysis of trace elements in humans. The elements detected for measurement were chosen based on their importance in biological processes. The importance of trace elements in clinical oncological science is well-established. Early detection and screening of precancerous disorders prevent the occurrence and prevalence of cancer in the general population.

Based on this perspective, the present study was designed to ascertain possible correlation of electrolyte profile of serum in normal and oral cancer volunteers. For this purpose, SELIBS analysis is used for rapid screening and elemental detection of human blood samples. Also, blood samples were collected from 24 oral cancer patients and 22 healthy volunteers to check the validity of the developed method for disease diagnosing. The differences in LIBS spectra between oral cancer patients and normal volunteers were compared to study the changes in serum electrolyte concentrations. The classification between normal and oral cancer groups was then investigated by using principal component analysis (PCA) and match-no-match analysis. The results obtained were encouraging for further research in this direction.

## 2 Experimental Section

### 2.1 Preparation of the Superhydrophobic Polydimethylsiloxane (PDMS) Substrate

The core objective of this work was to demonstrate the ability of the LIBS technique to detect “ultratrace elements” in biological fluids. Though many researchers have proposed interesting sampling approaches for liquid LIBS analysis, it has not been recognized as an efficient body fluid elemental analyzer to date. In the present study, the superhydrophobic substrate is fabricated via a two-step process, by replicating the laser-patterned master structure, which is free of complex experimental procedures, cost-effective, and easy to use. In practice, if we prepare such multiple substrates, we can very well perform body fluid analysis in situ or under conditions utilizing the portability of LIBS systems, which is not feasible using any of the lab based conventional techniques.

**Figure 1:**
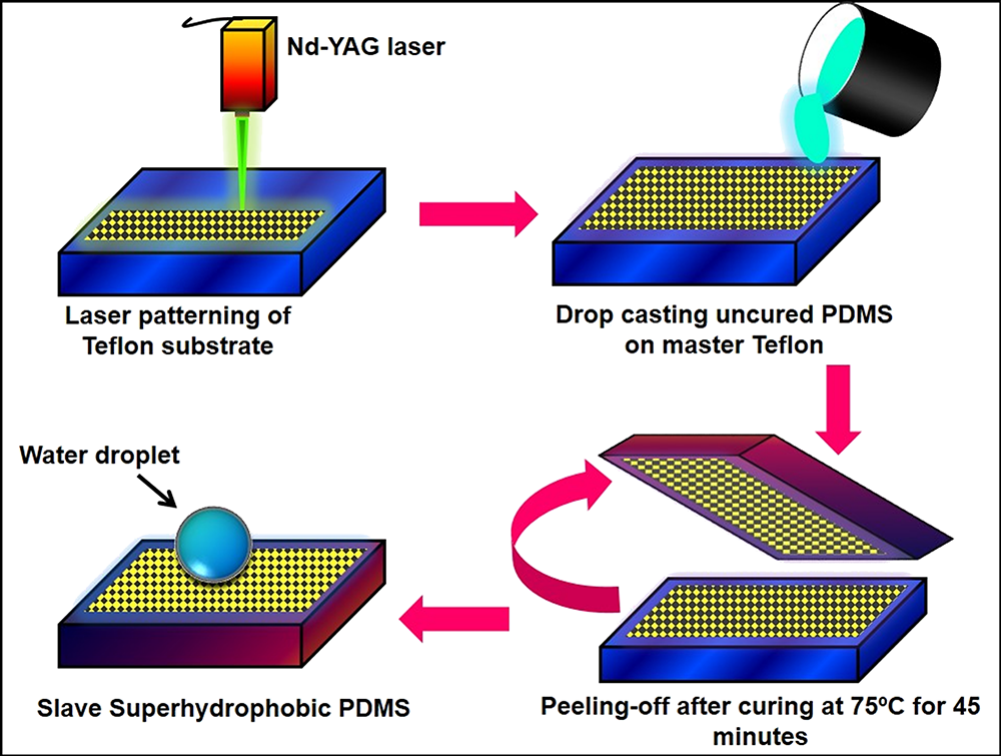
Schematic diagrams of the microreplica nanopatterns on PDMS film.

Nanosecond pulsed laser ablation process produces controlled roughness on metallic and polymeric surfaces via melting and solidification of the material. The fabrication process of PDMS replica from the patterned Teflon substrate is illustrated in Figure 1. This method helps create multiple structures on a polymer by replicating laser-created patterns from the master Teflon substrate to a soft elastomer like PDMS. This technique includes fabricating a master structure on a polymer and using this template to replicate its reverse pattern on the polymeric surface. The laser source used here is a Q-switched Nd:YAG pulsed nanosecond with a wavelength of 532 nm. This laser beam is directed to a 100 mm biconvex lens for delivering the laser beam over the sample surface. Laser patterns on the Teflon substrates are created by scanning the laser beam having 10 mJ energy and 10.64 μm focal spot size with different speeds and *y*-axis variations. Patterned Teflon substrates were cleaned by the sonication method using a mixed ratio of ethanol and DI water. The PDMS substrates were prepared using Sylgard 184 kit reported in our previous study.^23^ The thoroughly mixed and desiccated mixture of the prepolymer and curing agent (Sylgard-184A and Sylgard-184B) is carefully poured onto a cleaned patterned Teflon substrate. The solidified PDMS substrate is then peeled off and used for further studies.

### 2.2 Ethical Approval and Sample Collection

Ethical approval has been obtained from the Research Ethics Committee (REC), Manipal College of Dental Sciences (MCODS), Mangalore, for this study (Protocol Reference Number: 20101). Blood samples from oral cancer patients were collected, and sample collection procedures followed the ethical clearance guidelines regarding the informed consent of human volunteers. All of the subjects were informed about the study, and written consent was obtained from each one. Blood samples were collected from 22 healthy volunteers after informed consent. Samples were used as received for centrifugation. The schematic illustration of blood sample collection and processing is shown in Supplementary Figure 1. The serum and plasma samples were then carefully extracted from the upper part of the centrifuged sample using a pipet and transferred to sterile microcentrifuge tubes labeled with donor’s names and health status. The samples were stored at −80 °C for later LIBS analysis.

### 2.3 Laser-Induced Breakdown Spectroscopy (LIBS)

LIBS spectra were recorded by the developed direct coupled LIBS system discussed in the previous work.^24^ For this study, Cu, Cd, and Pb solutions were prepared with concentrations of 100, 600, and 100 ppb, respectively. 10 μL of the same was deposited onto the bare and superhydrophobic PDMS substrates for SELIBS analysis. Then, it was placed on the bare and superhydrophobic PDMS substrate and dried in an oven (80 °C), and the SELIBS activity was measured with a developed LIBS system. The LIBS signals were obtained with an average of the multiple single shots at each 10 μL dried sample spots. The energy of 8 mJ was used to excite the samples. The delay time and gate width were fixed at 900 ns and 300 ns, which have been optimized in previous work.^23^

### 2.4 Data Analysis

The recorded LIBS spectra were baseline-corrected and interpolated using GRAMS software (Thermo Scientific Inc., Rockford, IL). Principal component analysis (PCA) was done using the processed LIBS spectra of normal and oral cancer groups.^25^ Even chromatograms of the same sample recorded at different times and by different people may not be identical due to differences in sample handling, instrumental variations, environmental changes, and so on. Nonetheless, a given class of samples will have real similarity for a set of parameters, such as relative peak intensities, retention times, peak half widths, and so on. The Mahalanobis method is a powerful tool for determining the similarities between a set of parameters for an unknown test sample and the corresponding set of values for a calibration set of standard samples.^25^ When the unknown sample is predicted against various models (calibration sets), the material can be classified as belonging to the class with the closest match. The Mahalanobis method is extremely sensitive to intervariable variations in calibration data sets. We used it as a discriminating parameter in the match-no match technique. The Mahalanobis distance is a parameter used in the method.^26^ We do PCA analysis with the test sample added to the calibration set in order to determine the scores and residuals for the match/no match analysis of a test sample using the disease calibration set.

## 3 Results and Discussion

### 3.1 Surface Property of the Superhydrophobic PDMS Substrate

**Figure 2:**
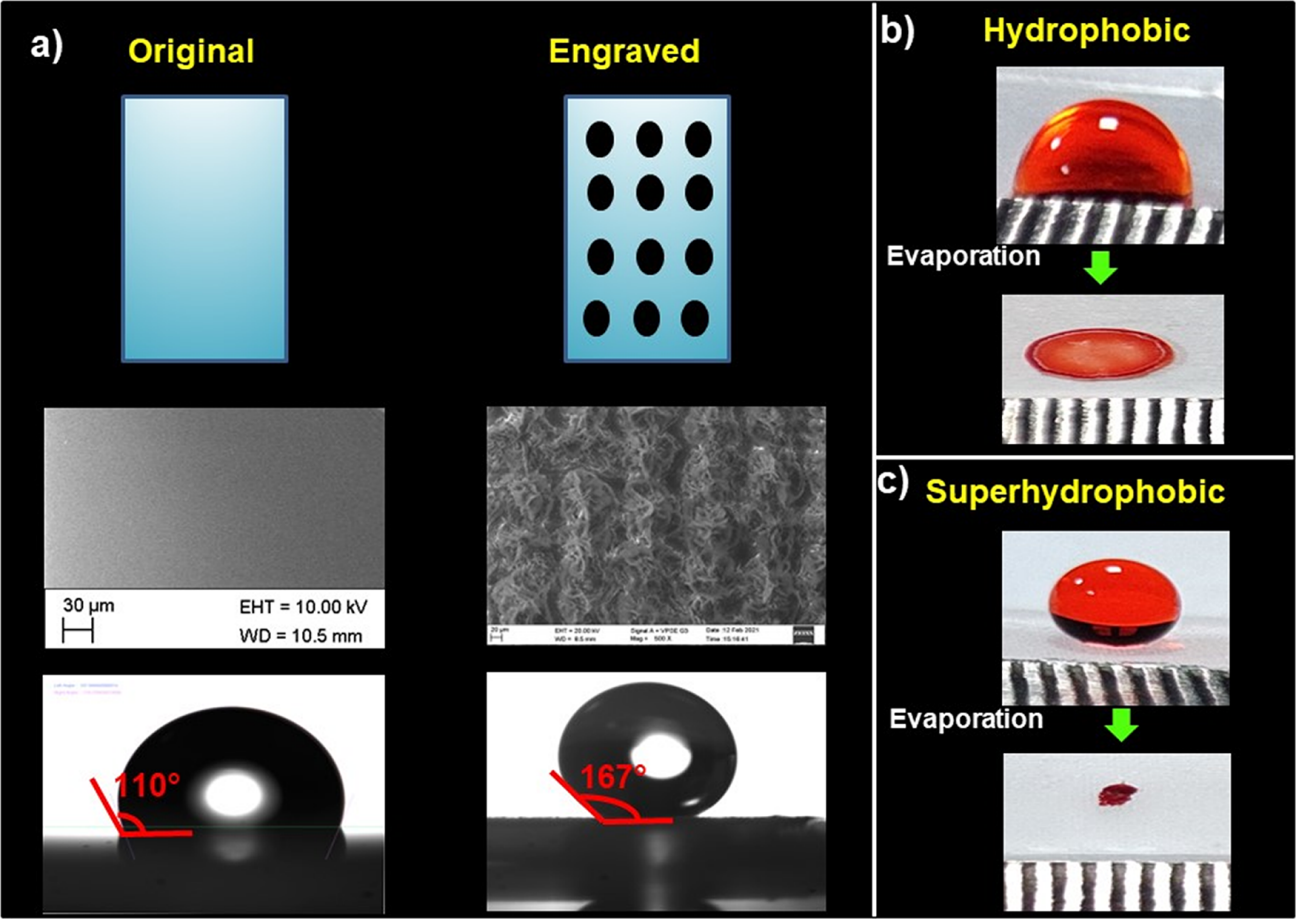
(a) SEM images of original PDMS and the patterned Teflon replica of PDMS and corresponding contact angle images; (b, c) evaporation of droplet on the bare and engraved Teflon replica of PDMS PDMS.

The surface smoothness of Teflon was changed by laser patterning followed by a soft lithography technique as discussed in experimental section 2.2. The typical contact angle measurements obtained under replicated PDMS substrates are shown in Figure 2a. As shown in Figure 2a, 10 μL of water exhibits a contact angle of ∼167° on the PDMS substrate, which is replicated from the patterned master Teflon substrate (negative replica), whereas it is nearly 110° for bare PDMS. To further explain the reason that the replicated PDMS substrate is better than the plane PDMS substrate on the ability to enrich analyte particles, the SEM images and evaporation profile are obtained and shown in Figure 2a–c. In Figure 2a for the original PDMS substrate, the surface shows a relatively smooth surface, but the master structure replicated area is embellished with microstructures, which turns PDMS into a superhydrophobic PDMS substrate. It shows that the surface is covered with microstructures as the negative replicas of the laser patterned Teflon substrate let the negative structure appear on the prepared PDMS replica. Figure 2 depicts a water droplet with food color that dries on the bare PDMS substrate and superhydrophobic PDMS substrate. After the evaporation process is finished, it creates a ring-shaped residue on bare PDMS with a diameter of 7 mm known as the coffee-stain effect. Once evaporation is completed, uniform particles are deposited in a nearly circular shape on the patterned PDMS substrate. It is clear from Figure 2b,c that most of the particles were deposited in the edges of the droplet, whereas particles are concentrated in small spots on super hydrophobic surfaces. The result reveals that superhydrophobic surfaces can provide a solution for achieving analyte enrichment in small area and also it could save time to prepare more number of SELIBS substrates. It is worth noting here that we collected signals from the ring wherein more particles are deposited in the bare (unpatterned) PDMS substrate, whereas the superhydrophobic PDMS with micronanostructures (patterned) could create densely packed analyte particles in a small area. As a result, the enrichment of analyte particles on the substrate led to a significantly enhanced signal and improved detection sensitivity.

### 3.2 Comparison of LIBS and SELIBS Studies

**Figure 3:**
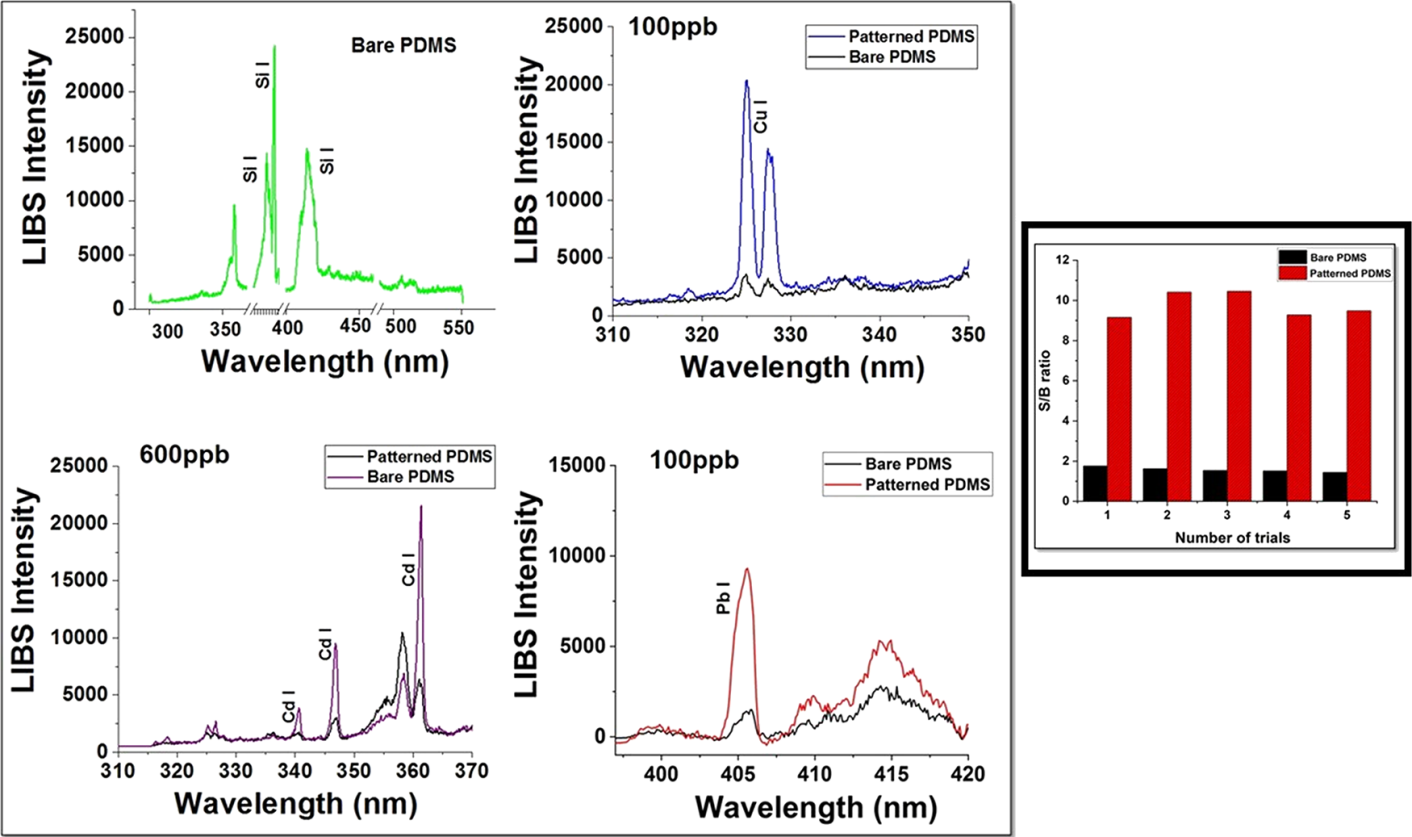
LIBS spectra of 100, 600, and 100 ppb Cu, Cd, and Pb on bare and patterned PDMS substrates. Inset: comprehensive comparison with the LIBS signals on the newly proposed superhydrophobic PDMS substrate with bare PDMS substrate to demonstrate the advantages of the new method using 100 ppb Cu.

A volume of 10 μL solution of the analyte of interest was dried on 5 spots and have been analyzed to study the improvement in the LIBS signal intensity. Once evaporation is completed, uniform particles are deposited in a nearly circular shape on the patterned PDMS substrate. The LIBS spectra were recorded on the ring, where the particles are deposited. We have taken the average of the multiple single shots at each 10 μL dried sample spots. To check the applicability of the prepared super hydrophobic PDMS substrate for trace elemental analysis, 10 μL of 100 ppb Cu solution was deposited onto the original and superhydrophobic PDMS substrate and dried using the oven. The first figure in Figure 3 shows the LIBS spectra of bare PDMS substrate, which is used for drying the analyte sample solution. Therefore, the distributions of analyte particles with concentrations of 100, 600, and 100 ppb were deposited on the bare and super hydrophobic PDMS substrate. The LIBS measurements were carried out on each of these spots and the results are shown in Figure 3.^20,23^ The LIBS signal intensity of Cu on the superhydrophobic PDMS substrate exhibits a huge enhancement in the LIBS signal as compared to the plane PDMS substrate. Figure 3 shows the comparison LIBS spectra of 10 μL of 100 ppb Cu on the bare and patterned PDMS substrate. The LIBS signal in the patterned PDMS substrate is enhanced 5 times for Pb and Cd and 9 times for Cu as compared to the bare PDMS substrate. It has been already known that Cu analyte solution evaporating on the bare PDMS substrate tends to spread particles on the larger area as compared with the superhydrophobic substrate. Therefore, the distribution of analyte particles on the original PDMS substrate is scattered and different from the center to the edge of the deposited area, contributing to the low reproducibility and the LIBS signal intensity, whereas the superhydrophobic PDMS with micronanostructures could achieve densely packed analyte particles in a small area, the enrichment of analyte particles on the substrate leads to a significant increase in the LIBS signal intensity. The analyte enrichment on the superhydrophobic substrate helps to enhance the LIBS signal (∼9 times in the case of 100 ppb Cu) intensity on the patterned PDMS substrate, which is evident from the Figure 3 inset.

### 3.3 Analytical Performance of the SELIBS Technique

**Figure 4:**
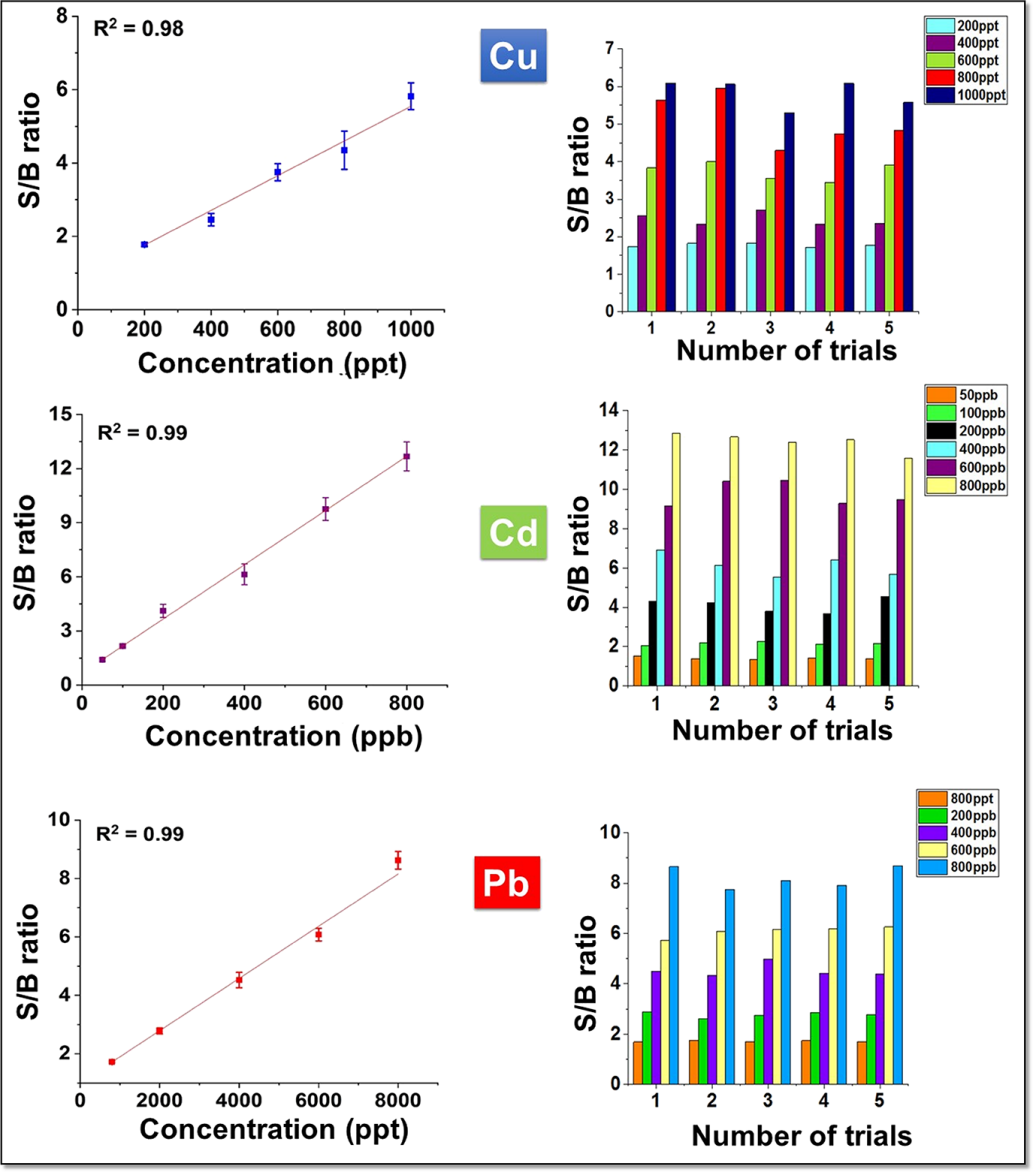
Calibration graphs of Cu, Cd, and Pb with the superhydrophobic PDMS substrate.

To check the sensitivity of the prepared superhydrophobic PDMS substrate, 10 μL of Cu with different concentrations was deposited and dried on the prepared PDMS replica, and their corresponding LIBS spectra were recorded. Figure 4 shows the linear correlation between the signal to background ratio and the different concentrations of Cu analyte. As for all the concentrations, the emission wavelength of Cu at 324.7 nm is observed, but the LIBS intensity of the Cu line declined with the decrease in analyte concentration.^19^ When the Cu concentration decreased up to 200 ppt, the LIBS signal could be still observed clearly, indicating high sensitivity of the prepared superhydrophobic PDMS substrate. As the analyte solution was further diluted (100 ppt), there was no identifiable peak from the obtained SELIBS spectra. It can be inferred that the detection limit of the as-prepared SELIBS substrate for Cu is 200 ppt. Also, we measured the LIBS spectra of Pb and Cd on developed substrates after evaporation of 10 μL of analyte solutions with different concentrations, which are shown in Figure 4. In short, the superhydrophobic PDMS substrate exhibited higher sensitivity and lower LOD than the bare PDMS substrate.^23^ Therefore, a superhydrophobic PDMS substrate was employed for analyte detection instead of bare PDMS in the following experiments.

### 3.4 Analysis of Human Blood Samples Using the Developed SELIBS Technique

**Figure 5:**
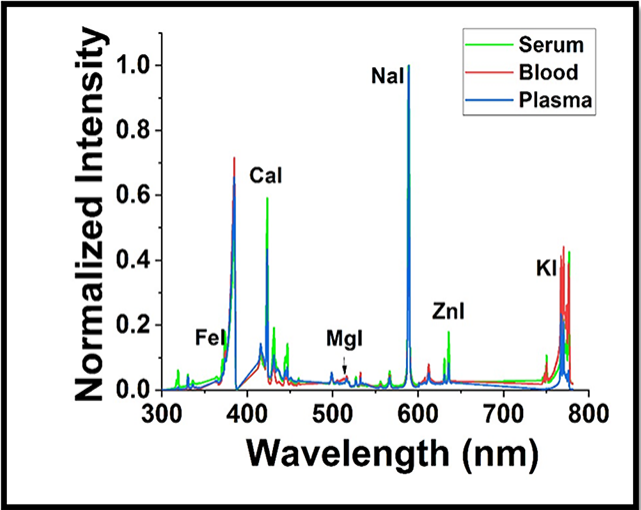
LIBS spectra of a blood sample using drop coating deposition

The goal of this study was to investigate the presence of trace elements in human blood, serum, and plasma samples. Among most of the elements, 10 of them are the major constituents of the body (C, H, N, Ca, P, K, Na, Cl, Mg, and S),^27^ 15 trace and ultratrace elements are considered essential for the human being, and only 7 have a well-established biological function (Fe, Cu, Zn, Se, Co, I, and Mo). Elements such as Cd, Pb, and Hg are well-known for their toxicity, whereas others (e.g., Rb or Sr) do not yet have their known roles. Serum and whole blood samples are the most commonly used primary samples in clinical laboratories. As discussed earlier, an unintentional disparity in the levels of trace elements can result in deficiencies or excessive amounts, leading to disorders. Our developed method enables the successive detection of the trace elements (K, Zn, Na, Fe, Ca, Mg) in both serum and whole blood using the said samples with volume as low as 10 μL only. Figure 5 shows the LIBS spectra of blood, serum, and plasma samples of a healthy volunteer. 10 μL of the sample was dried on a PDMS substrate, and the spectra were recorded in the region 300–800 nm. The highest LIBS intensity was observed for Na (588.9 nm) and Ca (422.67 nm) followed by K (766.49 nm), Fe (374.33), and Mg (518.36 nm), in the blood, serum, and plasma of normal volunteers. The LIBS spectra of blood, serum, plasma samples were normalized by dividing the highest intensity value observed for the sodium (Na) emission at 588.9 nm.

However, there was no significant difference between the trace elements in the blood, serum, and plasma samples.^28^ Further, the LIBS spectra were recorded from 5 females and 5 male normal volunteers, and the results are shown in Supplementary Figure 2. There were no significant differences in the elemental signatures of both these groups.

### 3.5 SELIBS Measurements for Oral Cancer Detection

In this study, the feasibility of the developed SELIBS technique for oral cancer detection was investigated. Monitoring the elemental changes in blood samples can help in the diagnosis, assessment, and treatment of oral cancer patients. However, these changes are often part of a more complex network of factors, and a comprehensive clinical evaluation is necessary for a complete understanding of a patient’s condition. Elevated levels of calcium in the blood may indicate hypercalcemia, a condition associated with oral cancer. Copper is an essential trace element in the body, but its levels need to be carefully regulated, and copper metabolism may be altered in cancer patients, including those with oral cancer. Variations in the emission line intensities of the Ca line in oral cancer and normal conditions were evaluated. LIBS data were obtained from 22 healthy subjects and 24 oral cancer patients based on drop coating deposition on the superhydrophobic SELIBS substrate (Table 1). Among them, we could analyze 20 normal and 18 diseased sample spectra after removing experimental and sample errors. Each LIBS measurement was repeated 10 times on separate spots and the average spectra were analyzed to extract further information.

**Table 1:** Number of Samples Collected for Biological Study

**group**	**no. of samples**	**gender**	**samples used**	**type of disease**
control	22	11 males	serum	
		11 females		
malignant	24	22 males	serum	oral cancer
		2 females		

**Figure 6:**
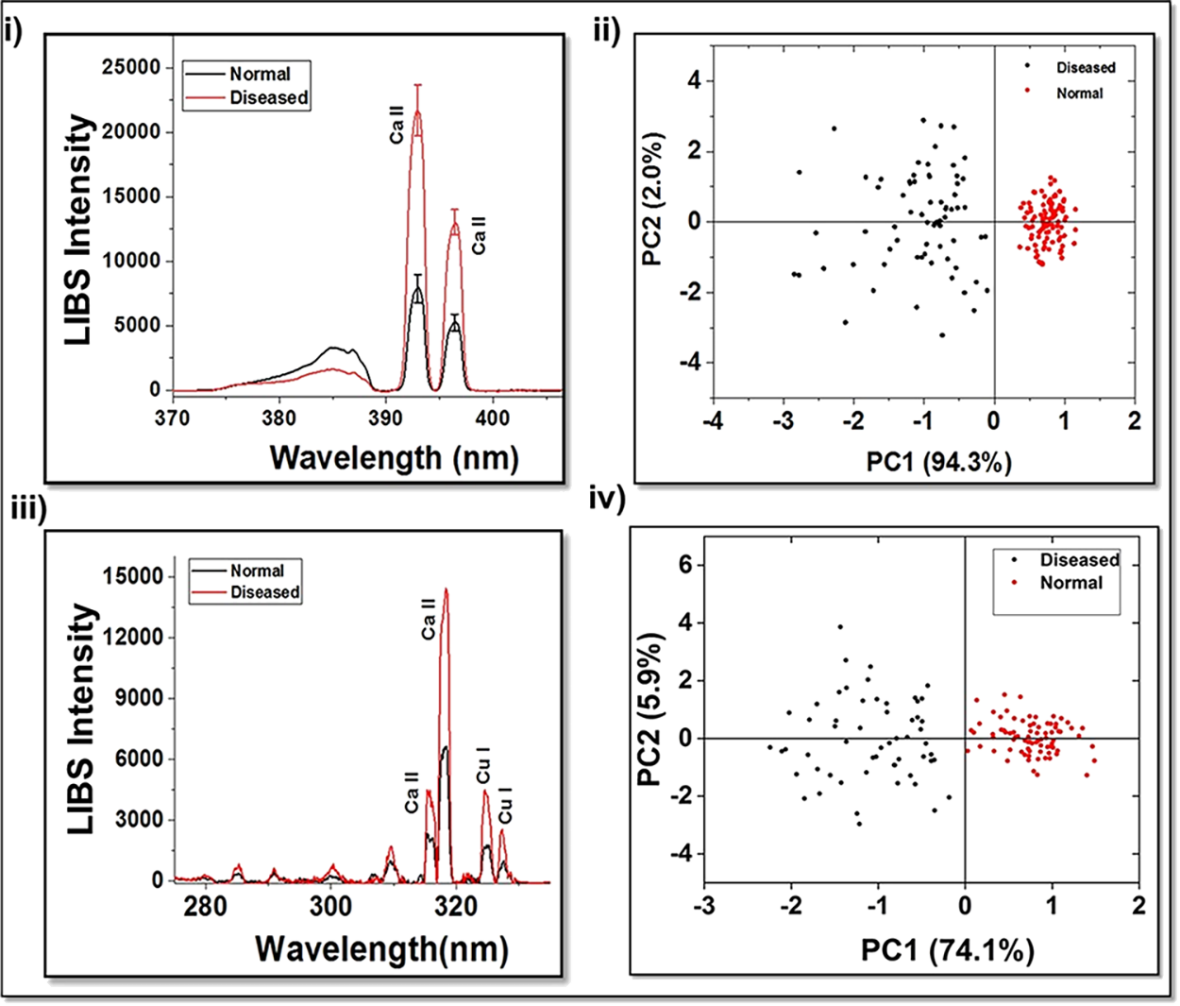
(i, iii) LIBS serum spectra indicating Ca and Cu variation in normal and oral cancer cases and (ii, iv) principal component analysis (PCA) for normal and diseased subjects – Ca, Cu variation.

Figure 6i describes the average LIBS spectra of the corresponding Ca emission lines from the two groups of serum samples. The main Ca emission peaks in the LIBS spectrum are also identified and assigned as shown in Figure 6 using the NIST spectral database. As shown, major spectral emissions were observed at 394 and 396 nm, respectively. Compared to the normal group, Ca II emission intensity was found to be increasing in the oral cancer cases.^29^ The feasibility of classifying normal and oral cancer groups using multivariate analysis from the acquired LIBS data was explored. The classification of the 38 serum samples was also carried out by the PCA method. The results showed that 100 spectra in the normal group and 90 spectra in the diseased group were classified into the respective groups accurately. The two-dimensional scatter plot diagram (Figure 6ii) of discrimination scores demonstrated a clear classification of these two groups. The present study reveals a marked Ca increase in oral cancer groups compared to normal. Serum calcium levels can be increased by local or generalized resorption of bone. Calcium in human serum is divided into three forms, including protein-bound calcium, calcium phosphate/citrate complexed calcium, and ionized calcium. The ionic calcium has physiological activity since it exists in its free form. Hypercalcemia symptoms are caused by abnormal concentrations of ionized calcium in the blood. Increasing serum Ca levels in oral cancer patients result from increased bone resorption, probably through interactions with parathormone receptors.^30^

As mentioned, compared with the serum samples of healthy individuals, the serum samples of those with oral cancer exhibited a significant increase in Cu levels. This was due to the high Cu levels in the areca nuts. Studies suggest that the higher levels of serum Cu found in cancer patients may be a result of an increased production of ceruloplasmin, a protein that contains Cu.^305^ The LIBS spectra of oral cancer and normal volunteers’ serum samples are shown in Figure 6iii. Intense Cu emissions were observed at the 324 nm region from oral cancer serum samples as compared to normal samples.

In order to develop and establish the classification and diagnostic models, PCA was employed for the said spectral region. The recorded LIBS spectra are analyzed and classified using PCA. Figure 6iv shows the score plot of the oral cancer and normal serum samples, clearly indicating good segregation of spectral data from normal and oral cancer volunteers. A preliminary attempt was made to assess these parameters as predictors of disease occurrence and progression. The analysis of LIBS data indicates that the variation of electrolyte in oral cancer patients, especially Ca and Cu, plays an important role in oral cancer serum samples.

A calibration set was created using LIBS data collected from oral cancer patients by monitoring the variation in the Ca and Cu elements in serum samples. This was followed by a match/no match test to distinguish between the data collected from oral cancer cases and normal. Subsequently, a separate principal component analysis (PCA) is conducted for these calibration sets, during which essential statistical metrics such as the “Mahalanobis Distance” and “spectral residuals” are computed for the individual volunteers of these standard sets. When we introduce a sample to be tested, it is integrated into the standard calibration set. Following this, PCA was executed again. The Mahalanobis Distance and spectral residual of the test samples were then compared to the parameters obtained from the standard set to determine whether the parameters of the test sample match or do not match those of the calibration set within a specified standard deviation range. The obtained results are shown in Table 2. This technique offers two significant advantages. First, it utilizes all of the factors derived from principal component analysis (PCA) that contribute to the data, as opposed to the limited two or three factors typically considered in the factor plot method. Second, and of greater importance, the decision-making process relies on statistical parameters that can be tailored to specific values. This allows decision-making to be based on the probability of the test sample’s association with the calibration set. The calculation of parameters such as sensitivity and specificity can then be performed using the results of the match/no match assessments. Consequently, decision-making becomes “observer-independent” and is grounded in the principles of Artificial Intelligence (AI) and Machine Learning (ML).

**Table 2:** Results of the Match/No Match Test Predicted against the Oral Cancer Calibration Set

**element**	**class**	**match (Count)**	**Mdistance range**
Ca	normal	yes (0)	
		no (97)	1.34–2.02
	diseased	yes (30)	
		no (4)	0.181–1.386
Cu	normal	yes (3)	
		no (65)	1.23–5.23
	diseased	yes (21)	
		no (3)	0.04–1.91

**Figure 7:**
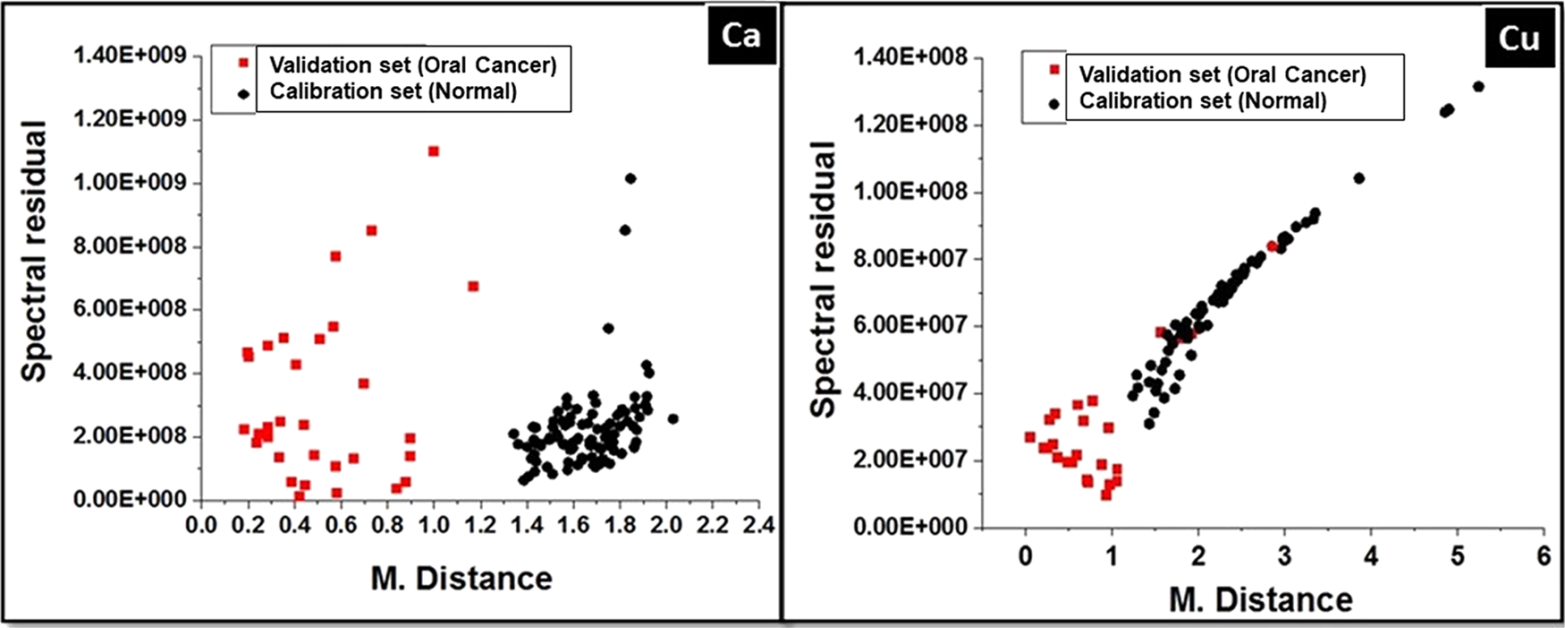
Mahalanobis distance vs spectral residual for an oral cancer standard set.

There is no match between any of the normal samples and the calibration set of oral cancer samples for Ca variation, while four of the oral cancer samples do not match the calibration set for oral cancer variation. For Cu variation, three of the normal samples matched the created standard set for oral cancer samples, while three of the oral cancer samples did not match the standard set. Despite the encouraging results for the oral cancer diagnosis, some data points do not match the expected oral cancer pattern, which is likely due to the cutoff value used for the match/no match test. The accuracy of the test was found by calculating sensitivity and specificity values.^31^ The studies on the variation of Ca and Cu showed a sensitivity above 87% and a specificity above 95%. Figure 7 shows the spectral residual vs Mahalanobis distance plot obtained from the match/no match test. It can be noticed that the Mahalanobis distance values for the samples that do not belong to the calibration set are high. We can use M distance values to determine the cut off points for inclusion of a sample in a specific class because M distance values are in units of standard deviation.

## 4 Conclusions

The nanosecond laser pattern method is proposed for fabricating roughness on Teflon and the PDMS substrate to create the superhydrophobic surface. The influence of nanosecond laser ablation parameters (translation speed and beam overlap) on the morphology and wettability of the resulting patterned replica was studied in detail. The uniaxial pattern on Teflon substrate gives a contact angle of more than 170°, but it is a very time-consuming process and expensive. Therefore, nanosecond laser patterned substrates were used to replicate the patterns onto the PDMS film through the soft lithography technique. It improves the surface property of the PDMS substrate to a superhydrophobic character. The process of removing the template from the replicated polymeric substrate has a significant influence on the final pattern’s roughness, shape, and size. Furthermore, we have demonstrated that the prepared superhydrophobic substrate is capable of elemental detection down to the parts per trillion level for Cu and Pb. The developed superhydrophobic PDMS substrate has been used for depositing and drying body fluids for the rapid detection of trace elements using LIBS. Normal and cancerous (oral cancer) serum samples were collected, and the LIBS signal was recorded. This is followed by multivariate analysis for classification. Results also indicate statistical interpretations using PCA and successful classification of the LIBS spectra. Spectral intensity differences are mainly observed in the Ca II emissions at 394 and 396 nm peaks and Cu emissions at 324.76 and 327.4 nm across the analyzed groups. Ca abundance was mainly observed in oral cancer samples due to the dissolution of bones and release of a large quantity of Ca into the blood. The match/no match test is performed using a calibration set created from LIBS data of oral cancer samples. The test results show 88% sensitivity and 96% specificity. The obtained result shows that the fabricated superhydrophobic PDMS substrates are efficient for detecting trace elements in biological samples efficiently, which is considered as the need of the hour.
